# *Lactobacillus helveticus* HY7801 Improves Premenstrual Syndrome Symptoms by Regulating Sex Hormones and Inflammatory Cytokines in a Mouse Model of Metoclopramide-Induced Hyperprolactinemia

**DOI:** 10.3390/nu16223889

**Published:** 2024-11-14

**Authors:** Hyeon-Ji Kim, Ji-Woong Jeong, Joo-Yun Kim, Jae-Jung Shim, Jae-Hwan Lee

**Affiliations:** R&BD Center, hy Co., Ltd., 22, Giheungdanji-ro 24 Beon-gil, Giheung-gu, Yongin-si 17086, Republic of Korea; skyatk94@gmail.com (H.-J.K.); woongshow@hy.co.kr (J.-W.J.); jjshim@hy.co.kr (J.-J.S.); jaehwan@hy.co.kr (J.-H.L.)

**Keywords:** premenstrual syndrome, probiotics, *Lactobacillus helveticus*, prolactin, cytokines

## Abstract

Background/Objectives: Premenstrual syndrome (PMS), a clinical condition that manifests in the form of various physical and psychological symptoms, occurs periodically during the luteal phase of the menstrual cycle and reduces quality of life. Methods: Here, we conducted in vitro and in vivo experiments to investigate the effects of *Lactobacillus helveticus* HY7801 (HY7801) on PMS symptoms. Results: Data from the in vitro experiments showed that HY7801 inhibits prolactin secretion by estradiol-induced GH3 cells, as well as the secretion of pro-inflammatory cytokines by LPS-induced Raw 264.7 cells. Additionally, the oral administration of HY7801 (10^9^ colony-forming units/kg/day) to mice with metoclopramide-induced hyperprolactinemia reduced uterine tissue mass and endometrial thickness, both of which were increased excessively in the presence of prolactin. HY7801 also regulated the serum levels of follicle-stimulating hormone and prostaglandin E1/E2, as well as recovering the progesterone/estradiol ratio. HY7801 also downregulated the serum levels of prolactin and pro-inflammatory cytokines such as interleukin (*Il*)-6, tumor necrosis factor-alpha (*Tnf*), and IL-1β. Finally, HY7801 reduced the expression of genes encoding inflammatory cytokines (i.e., *Tnf* and *Il-6*), cyclooxygenase-2 (*Cox-2*), and inducible nitric oxide synthase (*iNOS*) in mice with hyperprolactinemia. Conclusion: In summary, HY7801 may be a functional bacterium that alleviates PMS symptoms by modulating hormones and inflammatory markers.

## 1. Introduction

Premenstrual syndrome (PMS) refers to the unpleasant psychological, physical, and behavioral health changes that occur during the luteal phase of the menstrual cycle [1,2]. PMS begins 2 to 7 days before the start of menstruation, and disappears within 4 days of menstruation beginning [3]. More than 90% of women of reproductive age experience mild to severe PMS symptoms, with approximately 2–8% experiencing premenstrual dysphoric disorder [4,5]. Representative physical symptoms of PMS include breast pain, abdominal bloating, edema of the limbs, and headaches, while emotional symptoms include depression, anger, increased appetite, insomnia, anxiety, and tension [6,7,8]. Because PMS occurs repeatedly during each menstrual cycle, it is a long-term, chronic problem.

The causes and mechanism(s) underlying PMS have not yet been clearly identified; however, various hypotheses have been proposed, including an imbalance between estrogen and progesterone, increased prolactin levels, and sensitivity to prolactin and prostaglandins and neurotransmitters, as well as diet, genetic factors, and lifestyle [9,10,11,12,13]. According to recent studies, changes in circulating estradiol (E2) and progesterone levels, along with abnormal fluctuations in circulating sex hormones, during the menstrual cycle are associated with various PMS symptoms [14,15]. Also, the excessive secretion of prolactin causes PMS-associated symptoms such as weight gain, premenstrual breast pain, ovulation disorders, and, in many cases, abnormal immune responses [16,17,18]. Accordingly, previous studies have utilized hyperprolactinemic models as animal models for PMS experiments, in which excessive prolactin secretion was induced with metoclopramide (MCP) [18,19]. Furthermore, elevated prolactin levels induce inflammatory reactions that promote the release of prostaglandins [18,20]. Prostaglandins may also be the cause of vomiting, diarrhea, and headaches, which can be additional symptoms of PMS [17,21]. Therefore, research on therapeutic substances that improve PMS by controlling hormonal imbalances, as well as prolactin and prostaglandin levels, is needed.

The FAO/WHO defines probiotics as “live microorganisms, which when administered in adequate amounts confer a health benefit on the host” [22]. Recent studies show that probiotics exert a variety of physical and mental functions from the gut to the brain, affecting stress levels, anxiety, and depression [23,24,25]. Previously, we showed that *Lactobacillus helveticus* HY7801 (HY7801) improves vulvovaginal candidiasis in a mouse model by suppressing pro-inflammatory cytokine-related gene expression including tumor necrosis factor (TNF)α, interleukin (IL)-6, and IL-1β, as well as regulating inflammatory factors such as COX-2, iNOS, and NF-κB [26]. Additionally, an oral intake of HY7801 alleviated bacterial vaginosis by decreasing the number of *Gadrnella vaginalis* (GV) in the vaginal fluid, as well as the levels of vaginal cytokines, in GV-infected mice. In addition, HY7801 inhibited biofilm formation by GV, as well as the adhesion of the bacterium to epithelial cells [27]. However, despite evidence that HY7801 improves the symptoms of female reproductive disorders, its effects on PMS have not yet been studied.

The aim of the present study was to examine whether *Lactobacillus helveticus* HY7801, isolated from healthy Korean women, improves symptoms of PMS. First, we inferred the mechanism by which HY7801 alleviates PMS symptoms through conducting in vitro experiments to assess whether HY7801 directly affects prolactin secretion. We then examined the inhibitory effects of HY7801 on prolactin and pro-inflammatory cytokines in a mouse model of metoclopramide (MCP)-induced hyperprolactinemia. We also evaluated the regulatory effects of orally administered HY7801 on the secretion of sex hormones and prostaglandins in mice with hyperprolactinemia.

## 2. Materials and Methods

### 2.1. Sample Preperation

*Lactobacillus helveticus* HY7801 (HY7801) was isolated from healthy Korean women and stored in a −80 °C deep freezer. Bacterial stock contained MRS broth (BD Difco, Sparks, MD, USA) and 20% (*v*/*v*) glycerol. HY7801 was cultured at 37 °C for 24 h in MRS medium and then collected by centrifugation at 4000× *g* for 20 min. Pellets of HY7801 were washed twice and resuspended in sterile phosphate-buffered saline (PBS) for in vitro tests.

### 2.2. Cell Culture

The GH3 epithelial-like cell line, derived from the pituitary gland of a female rat with a tumor, was purchased from the American Type Culture Collection (ATCC; Manassas, VA, USA) and was maintained at 37 °C/5% CO_2_ in Kaighn’s Modification of Ham’s F-12 Medium (F-12K Medium; Gibco, Waltham, MA, USA) containing 15% horse serum (Gibco, Waltham, MA, USA), 2.5% fetal bovine serum (FBS; Gibco, Waltham, MA, USA), and 1% P/S (Gibco, Waltham, MA, USA).

Raw 264.7 macrophage cells were purchased from the ATCC and maintained at 37 °C/5% CO_2_ in Dulbecco’s Modified Eagle’s Medium (DMEM; Gibco, Waltham, MA, USA) containing 10% FBS (Gibco, Waltham, MA, USA) and 1% penicillin–streptomycin (P/S; Gibco, Waltham, MA, USA). Cells were used at passages 5–10.

### 2.3. Viability Assays of GH3 Cells

GH3 cells were cultured at 37 °C for 24 h in 48-well plates (5 × 10^4^ cells/well). After stabilization, the medium was replaced with F-12K without serum and antibiotics to instill starvation conditions. The cells were then treated with HY7801 (10^4^, 10^5^, or 10^6^ colony-forming units (CFU)/well) and were incubated for 24 h. Cell Counting Kit-8 solution (CCK-8; Dojindo, Kumamoto, Kyushu, Japan) was added to each for 3 h (37 °C/5% CO_2_), and the number of viable cells was analyzed by measuring absorbance at 450 nm in a BioTek^®^ Synergy HT Microplate reader (Santa Clara, CA, USA).

### 2.4. Measurement of Prolactin Secretion by GH3 Cells

GH3 cells were seeded into 24-well plates (1 × 10^5^ cells/well) and acclimatized for 24 h. Next, HY7801 (10^4^, 10^5^, or 10^6^ CFU/well) was added to the wells for 2 h, followed by the addition of 10 nM 17β-E2 (E2; Sigma-Aldrich, St. Louis, MO, USA) for 24 h. Next, the culture medium was collected and prolactin levels were measured using a Rat PRL/Prolactin ELISA Kit (LS-F3900, Lynnwood, WA, USA). The absorbance in each well was measured at 450 nm in a BioTek^®^ Synergy HT Microplate reader (Santa Clara, CA, USA).

### 2.5. Measurement of Pro-Inflammatory Cytokine Sercetion by Raw 264.7 Cells

Raw 264.7 cells were cultured in 12-well plates (1 × 10^5^ cells/well). After cell stabilization, the medium was replaced with fresh DMEM without FBS and P/S. Cultured cells were then pre-treated for 2 h with LAB strains (1 × 10^6^ CFU/well), followed by the addition of 1 μg/mL of lipopolysaccharide (LPS) for 18 h at 37 °C/5% CO_2_. The amounts of TNF and IL-6 secreted into the culture medium were measured using the BD OptEIA™ Mouse TNF Set and the Mouse IL-6 ELISA Set (BD 555268, BD 555240; BD Biosciences, San Diego, CA, USA).

### 2.6. Animal Experiments

Female ICR mice (14 weeks old, *n* = 28) were purchased from Central Lab Animal Inc. (Seoul, Republic of Korea). Mice were housed under constant ambient temperature (21~23 °C) and humidity (45~65%) conditions, and they were kept on a 12 h light/12 h dark cycle. After seven days of adaptation, mice were assigned randomly to one of four groups, each containing seven mice—non-treatment (CON); MCP (20 mg/kg/day); MCP + Prefemin (PFM, 100 mg/kg/day); and MCP + *L. helveticus* HY7801 (10^9^ CFU/kg/day). All mice, except for the non-treatment group, were injected intraperitoneally with MCP once every 2 days during the experiments. PFM and HY7801 were resuspended in 200 μL of saline and were administrated orally for 21 days. CON and MCP mice were orally fed the same volume of saline during the same period. Body weight and food and water intake were measured weekly. Blood, spleen, and uterine tissue samples were obtained at the end of the experiment. Collected blood was allowed to clot by standing at room temperature, followed by centrifugation at 3000× *g* for 20 min to obtain the serum samples. Separated serum and uterine tissue samples were stored at −80 °C. Some of the uterine tissue was used for histological analysis. All animal experiments were approved by the Institutional Animal Care and Use Committee, Hy Co., Ltd., Seoul, Republic of Korea (IACUC approval number: AEC-2024-0003-Y).

### 2.7. Measurement of Hormones and Pro-Inflammatory Cytokines in Serum

Separated serum samples were analyzed at LABISKOMA (Seoul, Republic of Korea) via the multiplex assay. Serum levels of prolactin, estradiol, progesterone, and follicle-stimulating hormone (FSH) were measured, in addition to serum levels of TNF-α, IL-6, and IL-1β. The levels of prostaglandin E1 (PGE1) and E2 (PGE2) in serum were measured using a Mouse PGE1/Prostaglandin E1 ELISA Kit and a Mouse PGE2/Prostaglandin E2 ELISA Kit (LS-F28568 and LS-F32354, LS Bio, Lynnwood, WA, USA), respectively. Absorbance at 450 nm was measured in a BioTek^®^ Synergy HT Microplate reader.

### 2.8. Histological Analysis of Uterine Tissue

The mass of uterine tissue collected from each mouse was measured immediately using a microbalance (Mettler Toledo, Columbus, OH, USA). Tissue samples were then fixed in 10% (*v*/*v*) formalin solution (Sigma-Aldrich), embedded in paraffin, sectioned, mounted on slides, and stained with hematoxylin and eosin (H&E) by DooYeol Biotech (Seoul, Republic of Korea). Images were taken with a Zeiss Axiovert 200M microscope (Carl Zeiss AG, Thornwood, NY, USA). Endometrial thickness was measured using Motic DSAssistant (Motic VM V1 Viewer 2.0).

### 2.9. Extraction of Total RNA and Gene Expression Analysis

Total RNA was extracted from uterine tissues using the Easy-spin Total RNA Extraction Kit (iNtRON Biotechnology, Seoul, Republic of Korea). Then, cDNA was synthesized at 37 °C for 60 min using the Omniscript Reverse Transcription Kit (Qiagen, Hilden, Germany). The synthesized cDNA samples were analyzed using the QuantStudio 6 Flex Real-time PCR System (Applied Biosystems, Foster City, CA, USA). Real-time PCR using the TaqMan^TM^ Gene Expression Master Mix (Applied Biosystems) and TaqMan Gene Expression Assays (Applied Biosystems) was conducted for gene expression analysis. The following genes were analyzed: glyceraldehyde-3-phosphate dehydrogenase (*Gapdh*, Mm99999915_g1), interleukin-6 (*Il-6*, Mm00446190_m1), tumor necrosis factor α (*Tnf*, Mm00443258_m1), cyclooxygenase-2 (*Cox-2*, Mm00478374_m1), and inducible nitric oxide synthase (*iNOS*, Mm00440502_m1). Expression levels of target genes were normalized to that of *Gapdh*.

### 2.10. Statistical Analysis

All data are presented as the mean ± standard error (SE). Differences between groups were evaluated via one-way analysis of variance (ANOVA), followed by Tukey’s post hoc test. All statistical analyses were conducted using GraphPad Prism 6.0 Software (GraphPad Software, San Diego, CA, USA); *p* < 0.05 was considered statistically significant.

## 3. Results

### 3.1. Viability of GH3 Cells

**Figure 1:**
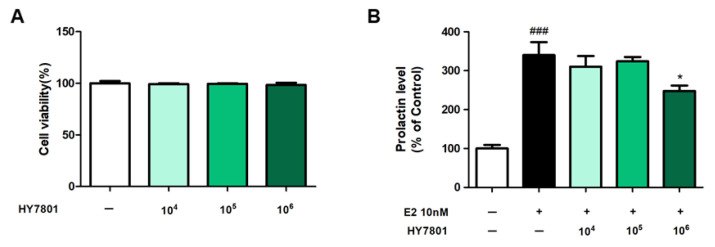
Effect of HY7801 on (**A**) the viability of GH3 cells and (**B**) the secretion of prolactin by estradiol (E2)-treated GH3 cells. Data are presented as the mean ± SE. ^###^ *p* < 0.001 vs. untreated group; * *p* < 0.05 vs. E2-treated group. E2: estradiol; HY7801: *Lactobacillus helveticus* HY7801 + E2.

CCK-8 was used to measure the effect of HY7801 on the viability of GH3 cells treated with different concentrations of HY7801 (Figure 1A). The survival rates of GH3 cells treated with 10^4^, 10^5^, or 10^6^ CFU/well of HY7801 were 99.15%, 99.62%, and 98.49%, respectively; thus, HY7801 did not affect the viability of GH3 cells at the concentrations tested.

### 3.2. Effects of HY7801 on Secretion of Prolactin by E2-Treated GH3 Cells

Next, we examined the effects of HY7801 on prolactin secretion by E2-induced GH3 cells (E2 plays a role in stimulating prolactin secretion) [28]. As shown in Figure 1B, the production of prolactin by E2-treated cells increased significantly to 340.12 ± 33.28% (*p* < 0.01) of that produced by untreated cells. Prolactin levels were 310.37 ± 37.50%, 324.29 ± 10.77%, and 247.68 ± 14.16% in medium from cells treated with 10^4^, 10^5^, or 10^6^ CFU/well of HY7801, respectively. HY7801 at 10^4^ and 10^5^ CFU/well caused a slight decrease in prolactin levels, but the reduction was not significantly different from that seen for E2-treated cells. HY7801 at 10^6^ CFU/well led to the significant suppression of prolactin levels relative to those in cells treated with E2 alone (*p* < 0.05). In other words, HY7801 exerts a suppressive effect on prolactin secretion.

### 3.3. Effects of HY7801 on Secretion of Pro-Inflammatory Cytokines by LPS-Induced Raw 264.7 Cells

**Figure 2:**
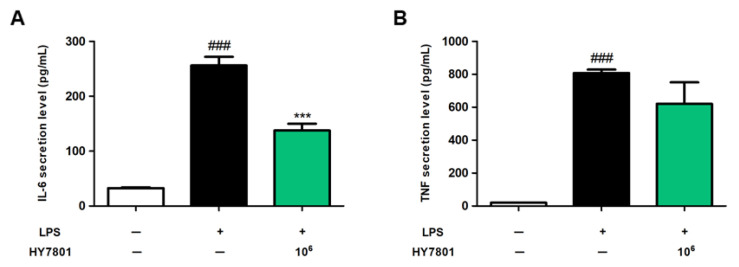
Effects of HY7801 on pro-inflammatory cytokines secreted by LPS-induced Raw 264.7 cells. (**A**) IL-6; (**B**) TNF-α. Data are presented as the mean ± SE. ^###^ *p* < 0.001 vs. untreated group; *** *p* < 0.001 vs. LPS-treated group. IL-6: interleukin-6; TNF-α: tumor necrosis factor-alpha; LPS: lipopolysaccharide; HY7801: *Lactobacillus helveticus* HY7801 + LPS.

Next, we investigated whether HY7801 affects the secretion of pro-inflammatory cytokines by LPS-induced macrophages. Figure 2A shows that untreated, LPS-treated, and HY7801-treated cells secreted IL-6 at 32.74 ± 1.45, 256.16 ± 15.60, and 137.60 ± 12.39 pg/mL, respectively. LPS-induced increases in IL-6 levels were suppressed significantly by treatment with HY7801 (*p* < 0.001). As illustrated in Figure 2B, the secretion of TNF-α by LPS-induced cells was significantly higher (807.54 ± 21.05 pg/mL, *p* < 0.001) than that by untreated cells (20.34 ± 1.13 pg/mL). In addition, HY7801 suppressed TNF secretion relative to that in LPS-induced cells, although the difference was not significant. Therefore, the data suggest that HY7801 downregulates the secretion of pro-inflammatory cytokines.

### 3.4. Effects of HY7801 on Physiological Indicators in MCP-Induced Mice

**Figure 3:**
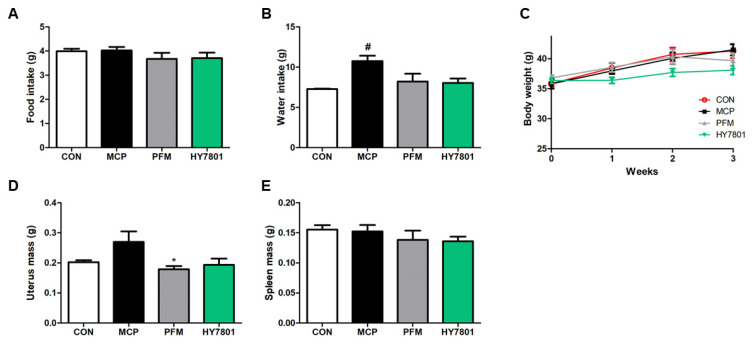
Effect of HY7801 on physiological indicators in mice with MCP-induced hyperprolactinemia. (**A**) Dietary intake; (**B**) water intake; (**C**) change in body weight; (**D**) mass of uterine tissue; and (**E**) mass of spleen tissue. Data are presented as the mean ± SE. ^#^ *p* < 0.05 vs. CON group; * *p* < 0.05 vs. MCP group. CON: non-treatment group; MCP: metoclopramide-induced mice; PFM: prefemin (100 mg/kg/day) + MCP; HY7801: *Lactobacillus helveticus* HY7801 (10^9^ CFU/kg/day) + MCP.

Mice with MCP-induced hyperprolactinemia were used to examine the effects of oral HY7801 on dietary intake, body weight, and mass of uterine tissue. PFM, a positive control, was used in this animal study (the main component of *Vitex agnus-castus* extract—lowers prolactin levels) [29]. First, we measured weekly food and water intake (Figure 3A,B). Dietary intake was similar between the groups; however, water intake by the MCP group was significantly higher than that by the control group (*p* < 0.05). Water intake by the PFM and HY7801 groups was similar to that by the control group.

Figure 3C shows that none of the mice in any of the groups demonstrated a significant change in body weight during the experiments. The mass of uterine tissue harvested from mice in the MCP-induced group (0.27 ± 0.035 g) was greater than that harvested from mice in the control group (0.201 ± 0.006 g), but the difference was not significant; however, the weight of uterine tissue harvested from mice in the PFM (0.179 ± 0.011 g) group was significantly lower than that harvested from mice in the MCP group (*p* < 0.05; Figure 3D). The amount of tissue harvested from the HY7801 (0.194 ± 0.021 g, n.s) group was also lower than that harvested from the MCP group, and was comparable with that harvested from the control group. The mass of spleen tissue harvested from the CON, MCP, PFM, and HY7801 groups was 0.155 ± 0.008, 0.152 ± 0.011, 0.138 ± 0.016, and 0.136 ± 0.008 g, respectively. The differences between the groups were not significant (Figure 3E).

### 3.5. Effects of HY7801 on Histological Analysis of Uterine Tissue

**Figure 4:**
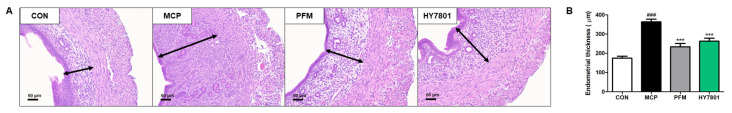
Histological analysis of uterine tissue from mice with MCP-induced hyperprolactinemia. (**A**) Hematoxylin and eosin-stained sections; 200× magnification; arrows point to the endometrium. (**B**) Endometrial thickness. Data are presented as the mean ± SE. ^###^ *p* < 0.001 vs. CON group; *** *p* < 0.001 vs. MCP group. CON: non-treatment group; MCP: metoclopramide-induced mice; PFM: prefemin (100 mg/kg/day) + MCP; HY7801: *Lactobacillus helveticus* HY7801 (10^9^ CFU/kg/day) + MCP.

Transverse sections of uterine tissue were stained with H&E (Figure 4A). Studies show that MCP increases the endometrial thickness index in mice [19]. As shown in Figure 4B, the endometrium in the MCP-treated group (363.74 ± 13.78 μm, *p* < 0.001) was significantly thicker than that in the control group (174.86 ± 10.11 μm). The administration of PFM and HY7801 led to a significant reduction in endometrial thickness, to 233.95 ± 16.72 μm and 263.02 ± 15.44 μm, respectively. The thickness of the endometrium in the HY7801 group was comparable with that in the PFM group.

### 3.6. Effects of HY7801 on Serum Levels of Sex Hormones

**Figure 5:**
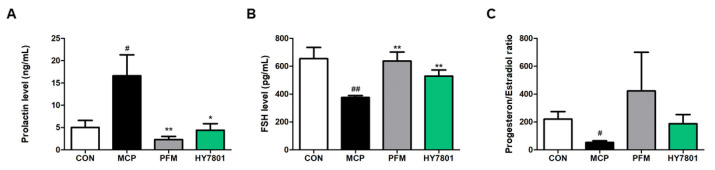
Effect of HY7801 on serum levels of sex hormones in mice with MCP-induced hyperprolactinemia. (**A**) Prolactin; (**B**) FSH; and (**C**) the progesterone/estradiol ratio. Data are presented as the mean ± SE. ^#^ *p* < 0.05 and ^##^ *p* < 0.01 vs. CON group; * *p* < 0.05 and ** *p* < 0.01 vs. MCP group. FSH: follicle-stimulating hormone; CON: non-treatment group; MCP: metoclopramide-induced mice; PFM: prefemin (100 mg/kg/day) + MCP; HY7801: *Lactobacillus helveticus* HY7801 (10^9^ CFU/kg/day) + MCP.

Next, to determine the effects of HY7801 on sex hormones, we assessed the levels of prolactin and FSH, as well as the progesterone/estradiol ratio (Figure 5A–C). Figure 5A shows that the levels of prolactin induced by metoclopramide were significantly higher than those induced by the control (*p* < 0.05). Prefemin (*p* < 0.01) and HY7801 (*p* < 0.05) led to significantly lower levels of secretion than in the MCP-induced group. FSH levels in the CON, MCP, PFM, and HY7801 groups were 654.07 ± 80.82, 375.91 ± 13.98, 637.75 ± 64.85, and 529.75 ± 44.05 pg/mL, respectively (Figure 5B). FSH secretion in the MCP-induced group decreased significantly (*p* < 0.01). Treatment with PFM restored FSH secretion to levels similar to those observed in the control group (*p* < 0.01). HY7801 also restored FSH levels compared with that in the MCP group, but the amounts were slightly lower than those in the PFM group. Figure 5C shows that the progesterone/estradiol (P/E) ratio in the CON, MCP, PFM, and HY7801 groups was 220.43 ± 53.85, 53.23 ± 11.48, 422.88 ± 277.33, and 187.93 ± 65.16, respectively; the differences between the MCP and PFM/HY7801 were not significant. However, the reduction in the P/E ratio induced by MCP was reversed by PFM and HY7801. In particular, HY7801 tended to recover the P/E ratio to a level similar to that in the control group. Thus, HY7801 may regulate sex hormones in mice with MCP-induced hyperprolactinemia

### 3.7. Effect of HY7801 on Serum Levels of Cytokines and Prostaglandin

**Figure 6:**
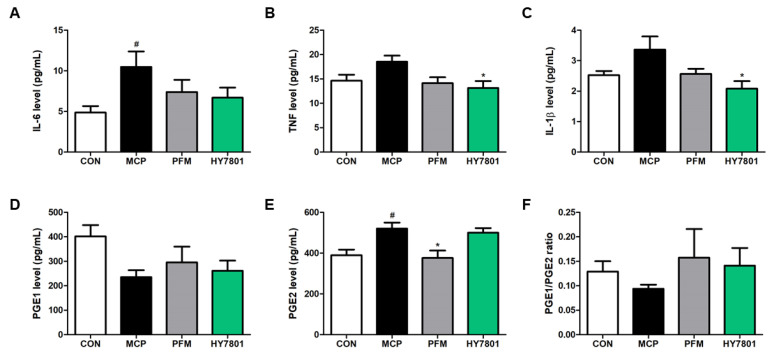
Effect of HY7801 on serum levels of pro-inflammatory cytokines and prostaglandin in mice with MCP-induced hyperprolactinemia. (**A**) IL-6; (**B**) TNF-α; (**C**) IL-1β; (**D**) PGE1; (**E**) PGE2 levels; and (**F**) the PGE1/PGE2 ratio. Data are presented as the mean ± SE. ^#^ *p* < 0.05 vs. CON group; * *p* < 0.05 vs. MCP group. IL-6: interleukin-6; TNF: tumor necrosis factor-alpha; IL-1β: interleukin-1β; PGE1: prostaglandin E1; PGE2: prostaglandin E2; CON: non-treatment group; MCP: metoclopramide-induced mice; PFM: prefemin (100 mg/kg/day) + MCP; HY7801: *Lactobacillus helveticus* HY7801 (10^9^ CFU/kg/day) + MCP.

Next, we investigated whether HY7801 affects the levels of pro-inflammatory cytokines such as IL-6, TNF, and IL-1β (Figure 6A–C). MCP increase the secretion of IL-6 to levels significantly higher than those in the control group (4.65 ± 1.90 pg/mL vs. 1.94 ± 0.79 pg/mL, respectively; *p* < 0.05). The increase in IL-6 secretion mediated by MCP was suppressed by PFM (3.67 ± 1.50 pg/mL) and HY7801 (3.04 ± 1.24 pg/mL), but the effect was not significant. The secretion of TNF in the MCP group (18.54 ± 1.24 pg/mL) was higher than that in the CON group (14.63 ± 1.23 pg/mL). HY7801 suppressed the levels of TNF significantly (13.14 ± 1.44 pg/mL) when compared with the MCP-induced group (*p* < 0.05). IL-1β levels showed a similar trend to TNF. The increase in IL-1β secretion induced by MCP injection (3.37 ± 0.43 pg/mL) was suppressed by HY7801 (2.08 ± 0.244 pg/mL, *p* < 0.05). Levels of TNF and IL-1β in the HY7801-treated group were lower than those in the positive control (PFM-treated) group.

We examined the effects of HY7801 on the prostaglandin levels induced by MCP injection (Figure 6D–F). PGE1 levels in the CON, MCP, PFM, and HY7801 groups were 401.74 ± 46.49, 235.35 ± 34.30, 295.54 ± 13.53, and 261.17 ± 41.72 pg/mL, respectively, although the differences were not significant; however, PGE1 levels in the PFM and HY7801 groups were slightly higher than those in the MCP group. In contrast, the PGE2 levels showed the opposite trend. PGE2 secretion in the MCP group (520.20 ± 29.57 pg/mL, *p* < 0.05) was higher than that in the CON group (389.95 ± 27.54 pg/mL). In addition, the level of PGE2 in the PFM group was significantly lower (376.35 ± 36.38 pg/mL, *p* < 0.05) than that in the MCP group. PGE2 levels in the HY7801 (500.22 ± 22.78 pg/mL) group were slightly lower than those in the MCP group, but the difference was not significant. Furthermore, the PGE1/PGE2 ratio in the CON, MCP, PFM, and HY7801 groups was 0.129 ± 0.021, 0.094 ± 0.009, 0.157 ± 0.015, and 0.141 ± 0.019, respectively, but differences were not significant; however, after the administration of PFM and HY7801, the reduction in the PGE1/PGE2 ratio induced by MCP injection recovered to levels similar to those in the control group.

Thus, HY7801 may alleviate PMS symptoms in MCP-induced model mice by suppressing the secretion of pro-inflammatory cytokines and regulating prostaglandin levels.

### 3.8. Effects of HY7801 on Gene Expression in Uterine Tissues

**Figure 7:**
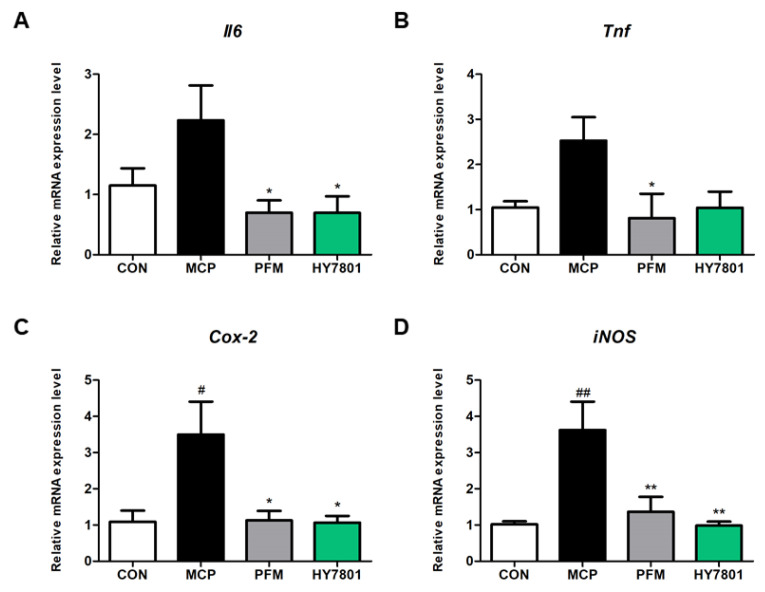
Effect of HY7801 on the expression of mRNA encoding inflammation-related genes in uterine tissues from MCP-induced hyperprolactinemia mice. (**A**) *Il-6*; (**B**) *Tnf*; (**C**) *Cox-2*; and (**D**) *iNOS*. Data are presented as the mean ± SE. ^#^ *p* < 0.05 and ^##^ *p* < 0.01 vs. CON group; * *p* < 0.05 and ** *p* < 0.01 vs. MCP group. *Il-6*: interleukin-6; *Tnf*: tumor necrosis factor-alpha; *Cox-2*: cyclooxygenase-2; *iNOS*: inducible nitric oxide synthase; CON: non-treatment group; MCP: metoclopramide-induced mice; PFM: prefemin (100 mg/kg/day) + MCP; HY7801: *Lactobacillus helveticus* HY7801 (10^9^ CFU/kg/day) + MCP.

Finally, we asked whether HY7801 affects the expression of inflammation-related genes and enzyme-related genes in uterine tissues. As shown in Figure 7A, the relative expression of mRNA encoding *Il-6* was higher (2.23-fold) in the MCP-treated group compared with that in the CON group (1.00-fold); however, *Il-6* levels in the PFM- and HY7801-treated groups were significantly lower (0.70-fold) than in the MCP group (*p* < 0.05). HY7801 reduced the expression of *Il-6* mRNA to a level similar to that in the positive control group. The expression of mRNA encoding *Tnf* was higher in the MCP group (2.53-fold) than in the CON group (Figure 7B), whereas treatment with PFM resulted in expression levels similar to those in the control group (0.81-fold, *p* < 0.05) and the HY7801 group (1.04-fold, n.s). The expression of *Cox-2* was 3.50-fold, 1.13-fold, and 1.07-fold in the MCP, PFM, and HY7801 groups, respectively (Figure 7C). The MCP-induced elevation in *Cox-2* expression was suppressed significantly by PFM and HY7801 (*p* < 0.05). As illustrated in Figure 7D, the increase in *iNOS* mRNA (3.62-fold, *p* < 0.01) after MCP induction was suppressed significantly via the oral administration of PFM (1.37-fold, *p* < 0.01) and HY7801 (0.98-fold, *p* < 0.01). Taken together, these results show that HY7801 downregulates the expression of mRNA encoding inflammation-related genes and enzyme-related genes.

## 4. Discussion

PMS is defined as a syndrome in which women experience various physical or psychological symptoms before menstruation [1]. Most (up to 90%) women of reproductive age experience PMS symptoms such as breast pain, abdominal bloating, headaches, depression, and anxiety, all of which affect family life, social life, and work performance; however, the mechanism underlying PMS remains unclear [30,31,32]. Recently, pharmacological treatments such as the inhibition of serotonin reuptake, gonadotropin-releasing hormone, and ovulation have been considered, but these may interfere with the production of sex hormones, causing side effects such as anovulation, decreased bone density, menopause, headaches, and depression [18,33]. Thus, women would benefit from natural alternative medicines. Previously, we demonstrated that the probiotic lactic acid bacterium *Lactobacillus helveticus* HY7801 (HY7801) improves vulvovaginal candidiasis and bacterial vaginosis [25,26]. In the present study, we conducted in vitro experiments to show that HY7801 reduces the secretion of prolactin and pro-inflammatory cytokines. In addition, we used a metoclopramide-induced mouse model to confirm that HY7801 reduces prolactin levels, regulates the levels of sex hormones and prostaglandins, and suppresses markers of inflammation.

Hyperprolactinemia is one of the main causes of PMS. Prolactin, a peptide hormone secreted by the pituitary gland, is secreted in response to eating, estrogen treatment, ovulation, and lactation. Prolactin also plays an essential role in metabolism, immune system regulation, and pancreatic development [9,34]. The excessive secretion of prolactin, a condition called hyperprolactinemia, may cause galactorrhea, infertility, and menstrual disruption in women [35,36]. In addition, abnormally high prolactin levels are associated with abnormalities in immune response [37]. First, we examined the effects of *Lactobacillus helveticus* HY7801 on prolactin secretion by E2-induced GH3 cells. E2, an estrogen steroid hormone, is a major female sex hormone that plays a role in inducing cell growth, as well as prolactin synthesis by GH3 cells [38]. This hormone plays a role in inducing cell growth as well as prolactin synthesis in GH3 cells [39]. Here, we show that HY7801 significantly suppressed E2-mediated increases in prolactin concentrations. We also found that the secretion of pro-inflammatory cytokines such as IL-6 and TNF-α was suppressed by HY7801. In other words, the in vitro data suggest that HY7801 has the potential to improve PMS by suppressing the hypersecretion of prolactin and cytokines.

To study the effects of HY7801 in vivo, model mice were injected intraperitoneally with MCP (20 mg/kg/day) for 21 days to induce hyperprolactinemia [18]. MCP increases prolactin levels in animals and humans, which, in turn, causes an imbalance between estradiol, progesterone, and FSH [19,40,41,42]. Prefemin, used as a positive control in this study, is a herbal medicine containing *Vitex agnus-castus* extract, which is used widely in Europe to treat women suffering from PMS [43]. MCP did not affect food intake, change body weight, or affect spleen mass; however, it did increase uterine tissue weight. Previous studies have predicted that the increase in uterine tissue weight may be due to an inflammatory response, and subsequent tissue edema, which is caused by prolactin or steroid hormones. [18,44,45]. The administration of PFM and HY7801 led to a significant reduction in uterine tissue mass compared with that in the MCP-induced group. We also found that PFM and HY7801 significantly reduced the prolactin concentration in the mice with MCP-induced hyperprolactinemia. In addition, histological analysis revealed that endometrial thickness was increased by MCP, but restored by PFM and HY7801. Thus, we propose that the decrease in endometrial thickness induced by HY7801 leads to a significant reduction in uterine tissue weight. In other words, the data suggest that the hypersecretion of prolactin induced by MCP is suppressed by PFM and HY7801, which decreases the thickness of the endometrium and reduces uterine tissue weight; however, further studies are needed to clarify the mechanisms underlying these results.

Hyperprolactinemia is associated with the suppression of FSH, which may affect the female reproductive organs and functions [41,46]. High prolactin levels interfere with the normal production of hormones such as estradiol and progesterone. Estradiol regulates prolactin production by acting directly on the pituitary. Thus, hyperprolactinemia affects the imbalance between estrogen and progesterone, which are related to the menstrual cycle [40]. Here, we found that serum FSH levels were reduced by MCP and recovered by HY7801. In addition, our data show that HY7801 restored the progesterone/estradiol ratio, which was decreased by MCP. These results indicate that HY7801 may regulate the imbalance in sex hormones caused by hyperprolactinemia.

Serum levels of pro-inflammatory cytokines IL-6, TNF, and IL-1β were lower in the HY7801 group than in the MCP-treated group. The expression of mRNA encoding *Il-6* and *Tnf* in the mouse uterus was also lower than that in the MCP group. These data are similar to the results of the experiments in LPS-induced Raw 264.7 cells, and suggest that the administration of HY7801 may alleviate abnormalities in the immune response induced by hyperprolactinemia.

Elevated prolactin concentrations induce inflammatory reactions that promote prostaglandin release [19,47]. Previous studies show that prostaglandins may contribute the pathophysiology and etiology of PMS [48,49]. Among the types of prostaglandins, those relevant to PMS are PGE1 and PGE2. PGE1 attenuates the biological actions of prolactin; therefore, low PGE1 levels exaggerate the effects of prolactin [10]. Thus, attempts have been made to treat PMS using an essential fatty acid precursor of PGE1. PGE2 is involved in female reproductive functions such as decidualization, ovulation, implantation, and pregnancy [50]. In contrast to PGE1, PGE2 is induced by prolactin [19]. In particular, PGE2 causes inflammatory and neuropathic pain, as well as nausea, vomiting, fever, and excessive uterine contractions, all of which are symptoms of PMS [51]. We confirmed that HY7801 reversed the reduction in the PGE1 and PGE2 concentrations caused by MCP. The administration of HY7801 restored the PGE1/PGE2 ratio to the level observed in the control group.

Finally, we investigated the additional inflammatory responses induced by HY7801 in the hyperprolactinemia mouse model by analyzing the expression of mRNA encoding *Cox-2* and *iNOS*. Cox converts arachidonic acid to prostaglandin. Cox-2 is expressed in response to inflammatory factors and growth factors, as well as other physiological stimuli, and plays a role in the production of prostaglandins that mediate pain and support the inflammatory process [52]. iNOS is a product of typical inflammatory processes, and the expression of iNOS is accompanied by the release of other mediators such as PGE2 and prostacyclin, driven by the Cox pathway [53,54]. Furthermore, the overexpression of iNOS is induced in response to TNF, IL-6, and IFN-γ [55]. Here, we found that the elevated expression level of *Cox-2* and *iNOS* genes mediated by MCP was reduced significantly by HY7801. Taken together, our data indicate that HY7801 alleviates the symptoms of PMS by suppressing inflammatory pathways. This study confirmed the improvement in efficacy of the administration of probiotic strain HY7801 on prolactin and inflammatory markers, which are known to be the causes of PMS symptoms. Nevertheless, a limitation of this study is that the molecular mechanism for the effect of HY7801 intake on systemic sex hormone changes was not confirmed. Therefore, further studies are needed to investigate the role of the probiotic HY7801 in the gut microbiota of the PMS model, as well as the metabolic changes and correlation between PMS symptom-related indicators and intestinal flora.

## 5. Conclusions

Here, we examined the effect of *Lactobacillus helveticus* HY7801 on PMS symptoms both in vitro and in an animal model. The in vitro experiments showed that HY7801 suppresses the secretion of prolactin by E2-induced GH3 cells, as well as the secretion of pro-inflammatory cytokines by LPS-induced Raw 264.7 cells. HY7801 showed the same activity in mice with MCP-induced hyperprolactinemia. Furthermore, the data suggest that HY7801 improves PMS symptoms by regulating sex hormone levels, prostaglandin levels, and the expression of genes encoding inflammatory markers. Therefore, we propose that HY7801 be used as a functional supplement that improves PMS symptoms and improves female health. In a further study, we plan to analyze changes in metabolites and gut microbiota in a PMS model following HY7801 intake to investigate the PMS alleviation mechanism.

## Data Availability

The data presented in this study are available in the article.
